# Optimising Hand Trauma Management in a Tertiary National Health Service (NHS) Plastic Surgery Department: A Quality Improvement Initiative Aligned With the United Kingdom’s Prevalence Data and British Society for Surgery of the Hand (BSSH) Guidelines

**DOI:** 10.7759/cureus.92661

**Published:** 2025-09-18

**Authors:** Shimul Dey, David Izadi

**Affiliations:** 1 Plastic and Reconstructive Surgery, University Hospital Coventry and Warwickshire National Health Service (NHS) Foundation Trust, Coventry, GBR

**Keywords:** bssh standards, hand trauma management, nhs guidelines, optimal surgical timing, quality improvement projects

## Abstract

Background: Hand trauma constitutes a significant proportion of emergency department presentations in the United Kingdom, leading to considerable functional impairment and imposing substantial healthcare burdens. The British Society for Surgery of the Hand (BSSH) provides critical guidelines for the timing of surgical intervention, aiming to optimise patient outcomes. This paper evaluates compliance with BSSH guidelines within a tertiary National Health Service (NHS) Plastic Surgery Department, correlating local findings with UK-wide prevalence data to identify systemic challenges and propose solutions to reduce the clinical and economic burden.

Methods: A retrospective observational audit, representing the second cycle of a quality improvement initiative, was conducted at University Hospitals Coventry and Warwickshire (UHCW) NHS Trust. Data from 148 hand trauma cases managed between January 1st and March 31st, 2025, were analysed. The primary objective was to assess the adherence of surgical interventions to established BSSH timeframes: within 24 hours for open fractures and open joints, bites within 24 hours, within four days for other open hand injuries, and within seven days for closed hand fractures. The first cycle audit (August 2022-May 2023) formed part of the national BSSH-RSTN collaborative audit.

Results: The audit revealed an encouraging overall compliance rate of 83% (82 of 99 eligible cases) for surgical intervention, successfully exceeding the project's 80% target and demonstrating an improvement from the 73% observed in the first cycle. While open soft tissue injuries showed high compliance (average 89-97%), persistent challenges were noted for open fractures (64% (9 of 14 cases) compliance) and particularly for closed hand fractures (67% (16 of 24 cases) overall compliance, with surgical fixation at 50% (7 of 14 cases). The median time from the decision to treat to surgical intervention was 42.7 hours.

Conclusion: Despite notable progress in overall compliance, persistent delays in the management of complex injuries such as open and closed fractures highlight ongoing systemic bottlenecks within the hand trauma pathway. Strategic interventions, derived from departmental Quality Improvement and Patient Safety (QIPS) discussions, are essential to further enhance patient outcomes and mitigate the substantial functional and economic burden of hand trauma in the UK, estimated at approximately £287 million annually. Key recommendations include increasing dedicated trauma list capacity, enhancing Minor Injuries Unit (MIU) and 52 Pre-Op (daily plastics trauma clinic) capabilities, implementing targeted education for referring units, and establishing regular rolling audits with comprehensive documentation.

## Introduction

Hand injuries account for 19-23% of all trauma-related emergency attendances in the UK, with over 1.2 million cases annually [1]. These injuries predominantly affect the working population and carry significant direct and indirect costs due to functional impairment, prolonged rehabilitation, time off work, and medicolegal implications [2,3]. The complexity of hand anatomy, including multiple tendons, nerves, joints, and small bones within a confined space, requires precise and time-sensitive management.

Recognising this, the British Society for Surgery of the Hand (BSSH) has established clear timeframes for surgical management, based on strong pathophysiological evidence showing time-sensitive risks of infection, scarring, and impaired healing. The specific guidelines for our study are as follows: 1) open hand fractures or joints: within 24 hours, 2) bite wounds: within 24 hours, 3) other open hand injuries (e.g., tendon, nerve): within four days, 4) closed hand fractures: within seven days [4].

These guidelines are based on strong pathophysiological evidence showing time-sensitive risks of infection, scarring, and impaired healing. This audit aims to assess UHCW’s compliance with these standards during the second audit cycle and to identify key systemic bottlenecks with the goal of improving patient care.

## Materials and methods

This retrospective local audit, representing Cycle 2 of a quality improvement initiative, analysed data from January 1 to March 31, 2025, at University Hospitals Coventry and Warwickshire (UHCW) NHS trust. UHCW is a tertiary centre with a dedicated Plastic Surgery Department, serving a significant regional population. The first cycle of this audit was conducted from August 2022 to May 2023 as part of a national initiative focused on improving hand trauma care quality and compliance with BSSH guidelines, providing a crucial baseline for comparison and evaluation of progress.

Inclusion criteria

The study cohort included all acute hand trauma patients who were treated by the Plastic Surgery Department at UHCW between January 1 and March 31, 2025, and met the specific criteria for surgical intervention as per the BSSH guidelines. During the data analysis, only cases that required surgical intervention were tabulated and calculated for compliance.

Exclusion criteria

Patients managed by the orthopaedic trauma and hand team as per trust protocol (e.g., all wrist and distal radius fractures, and based on the postcode system) were excluded. In our trust, hand trauma patients are allocated to either the plastic surgery or orthopaedic hand team based on a postcode system. However, all patients requiring revascularisation, regardless of postcode, are treated by the plastic surgery team. Inpatient referrals are managed conservatively, while MIU patients without a documented follow-up plan, Cases involving post-surgical complications or chronic hand problems and those managed solely through virtual review were also excluded.

Data were meticulously extracted from electronic patient records (EPR) and theatre logs. This involved a systematic review of patient notes to identify all relevant hand trauma cases managed within the specified timeframe. Key variables were carefully collected and analysed to assess various aspects of patient care and compliance.

The specific variables extracted included

Type of Injury

Each case was categorised by injury type, distinguishing between open and closed injuries, and further specifying the presence of fractures, tendon involvement, nerve damage, or signs of infection. This detailed classification was crucial for assessing compliance against specific BSSH guidelines, as different injury types have distinct recommended surgical timeframes.

Time From Injury to Presentation and Decision to Treat

These timestamps were critical for understanding potential delays in the initial patient journey, from the moment of injury to the patient's arrival at a healthcare facility and the subsequent clinical decision to proceed with surgical intervention. This helped identify pre-hospital or early hospital-phase bottlenecks.

Time to Definitive Surgical Management

This metric, recorded from the decision to treat until the actual surgical procedure, served as the primary measure for BSSH guideline adherence. It directly reflects the efficiency of the surgical pathway.

Treatment Location

The setting where the patient received definitive treatment was recorded to identify potential bottlenecks or efficient pathways. Specifically, "52 Pre-Op" refers to the daily Plastics Trauma Clinic, "DSU" denotes the Day Surgery Unit, "CEPOD" indicates the Emergency Theatre List used for urgent cases, and "MIU" represents the Minor Injuries Unit. Understanding where patients were treated helped to assess resource utilisation and capacity.

Anaesthesia Type

The type of anaesthesia used (e.g., local, regional, or general) was also noted, although its direct impact on BSSH compliance was not a primary outcome of this audit cycle.

Compliance With BSSH Guidelines

Compliance was rigorously assessed against established BSSH timeframes. It was measured as per the BSSH Guidelines for standardised patient care for that particular trauma. This was done in conjunction with local trust guidelines for first aid and the administration of appropriate antibiotics, which was ensured as per trust policy. Surgical intervention was deemed compliant if it occurred within 24 hours for open fractures, open joints, and bite wounds; within four days for other open hand injuries (such as tendon and nerve); and within seven days for closed hand fractures. Cases that exceeded these specified windows were categorised as non-compliant, allowing for a clear quantitative measure of adherence to national standards.

## Results

Summary

Within the study period, a total of 148 hand trauma cases were identified. Of these, 122 patients presented to a healthcare professional on the same day of injury, and 100 were seen by the Plastic Surgery team at UHCW within 24 hours. Surgical intervention was performed in 99 cases, representing 67% of the total identified cases. The overall compliance rate with BSSH guidelines for surgical intervention was 83% (82 of 99 cases), successfully exceeding the 80% target proposed for the project. This represents a significant improvement from the 73% compliance observed in the first audit cycle [5]. The median time from the decision to treat to surgical intervention was calculated as 42.7 hours. The audit confirms that hand injuries continue to represent a substantial workload, with a male-to-female ratio of approximately 2:1 and a mean age of 43 years, consistent with national data showing a high incidence in the working-age population [6].

Patient cohort

The study identified a total of 148 hand trauma cases. The patient cohort showed a male-to-female ratio of approximately 2:1, with a mean age of 43 years. The age range was from the youngest patient, 8 years old, to the oldest patient, 99 years old. The majority of cases (115 of 148, 77.8%) occurred in the 15-60-year-old age group, which aligns with national data showing a high incidence in the working-age population.

The common causes of these injuries were found to be work-related incidents, accounting for 21% (31 of 148 cases); home injuries, representing 19.5% (29 of 148 cases); sports-related incidents, making up 19% (28 of 148 cases); and falls, contributing to 15% (22 of 148 cases) of the total.

Treatment settings

Patients were treated across various settings within the department. The 52 Pre-Op clinic, which functions as the daily Plastics Trauma Clinic, managed 47% (47 of 99 cases) of cases. The Day Surgery Unit (DSU) handled 38% (38 of 99 cases) of interventions, while the Minor Injuries Unit (MIU) was utilised for 8% (8 of 99 cases) of cases. The remaining 7% (7 of 99 cases) of cases were managed via the Emergency Theatre List (CEPOD).

Compliance by injury type

Analysis of compliance by injury type revealed varied adherence to BSSH targets, which has been summarised in Table 1. Open soft tissue injuries demonstrated excellent compliance, with 97% (32/33) of cases meeting the ≤4 days target. Notably, all 10 bite wound cases met the stricter ≤24 hours target, achieving 100% compliance. Additionally, one patient requiring wrist-level revascularisation was taken to theatre within 2 hours of injury, highlighting excellent adherence for time-critical, limb-threatening cases.

**Table 1:** Comparison of UHCW Hand Trauma Surgical Compliance with BSSH Standards (Second Cycle Audit, Jan-Mar 2025) Median time to surgery: 42.7 hours; Overall compliance: 83% (82/99) (Cycle 2), up from 73% in Cycle 1 BSSH: British Society for Surgery of the Hand, UHCW: University Hospitals Coventry and Warwickshire

Injury Type	BSSH Target	UHCW Compliance (n/total)
Open soft tissue	≤4 days	97% (32/33)
Bite wounds	≤24 hours	100% (10/10)
Open fractures/joints	≤24 hours	64% (9/14)
Tendon injuries	≤4 days	76.5% (13/17)
Closed fractures	≤7 days	67% (16/24)

Challenges persisted for open fractures and joints, where only 64% (9/14) of cases met the ≤24 hours target. Tendon injuries showed 76.5% (13/17) compliance against their ≤4 days target, and closed fractures had 67% (16/24) overall compliance with the ≤7 days target, with surgical fixation specifically at 50% (7/14).

Of the 148 total cases, 122 patients presented to a healthcare professional on the same day of injury, and 100 were seen by the Plastic Surgery team at UHCW within 24 hours. The mean time from initial presentation at A&E to the decision to treat was 30.6 hours. The median time from the decision to treat to surgical intervention was calculated as 42.7 hours.

Overall, the compliance for Cycle 2 was 83% (82/99), successfully exceeding the project's 80% target and demonstrating an improvement from the 73% observed in the first cycle.

## Discussion

Pathophysiological rationale for timely intervention

Timely surgical intervention in hand trauma is critical due to well-established pathophysiological principles that directly impact patient outcomes.

Open fractures and joints within 24 hours

Open fractures are highly susceptible to contamination. Bacterial colonisation, particularly by *Staphylococcus aureus*, typically begins within 6-12 hours [6]. Delaying debridement beyond 24 hours significantly increases the risk of deep infection, osteomyelitis, and septic arthritis, especially in joints where synovial fluid lacks intrinsic immune defence [6]. Studies indicate that infection rates may rise significantly when intervention is delayed beyond this critical 24-hour window [7]. This emphasises the urgency required for these types of injuries to prevent severe, long-term complications.

Other open injuries within four days

Delayed repair of other open injuries, such as tendon or nerve lacerations, can lead to irreversible complications. For tendons, fibrin matrix formation begins within 48-72 hours, which can impair tendon glide mechanics and lead to adhesions, significantly compromising functional recovery [4,8]. In nerve injuries, prolonged denervation can result in irreversible muscle atrophy, making successful reinnervation more challenging [4]. Furthermore, contaminated wounds face increased infection rates if not thoroughly debrided and closed within this timeframe [6].

Closed fractures within seven days

While less immediately critical than open injuries, timely management of closed fractures is also important. Early callus formation typically begins around day 5-7 [6], which can complicate anatomical reduction if surgery is delayed, potentially increasing the risk of malunion or non-union. Additionally, fracture blisters may form after 48 hours, which can delay surgery and raise complication rates, impacting the overall healing process and patient comfort [7,9]. Studies have shown that delaying surgery for distal radius fractures beyond two weeks can lead to poorer patient-reported outcomes [10].

Overall performance and comparison with the first cycle 

This second audit cycle demonstrates significant improvement in overall guideline adherence (83% [82/99] vs. 73% in Cycle 1), particularly for open soft tissue wounds. However, persistent delays in managing closed fractures (67% [16/24] compliance) and tendon injuries (76.5% [13/17]) remain consistent with national findings reported in the BSSH-RSTN collaborative audit [5], indicating systemic challenges requiring further targeted intervention.

Pathophysiological implications of delayed care

The 36% (5 of 14 cases) non-compliance rate for open fractures represents a significant clinical concern. As established by Angermann & Lohmann [6] and supported by recent BSSH standards [4], each hour of delay increases the risk of bacterial colonisation, biofilm formation, and subsequent deep infection or osteomyelitis. The median delay of 37 hours for these injuries places patients at substantial risk for long-term complications [6,7,11]. The 67% (16 of 24 cases) compliance for closed fractures indicates considerable room for improvement. While the median delay of 112 hours approaches the seven-day target, frequent exceedances are problematic. Beyond this timeframe, soft callus formation and haematoma organisation complicate anatomical reduction, increasing the risk of malunion and poorer functional outcomes [7,9] as emphasised in BSSH guidelines [4]. Research has shown that delayed fixation can lead to increased rates of digit stiffness and lower wrist motion [10].

System-level challenges

Our analysis revealed two primary categories of systemic challenges: "front-end" delays related to initial patient pathways and "back-end" issues with surgical capacity.

Initial pathway delays

Our data indicates that while most patients present promptly to a healthcare facility, there is often a significant delay before they are seen by the specialist team. This can be attributed to referral pathway inefficiencies, where a patient seen at a Minor Injuries Unit (MIU) or Emergency Department (ED) experiences a delay while awaiting transfer or specialist review. A more granular cause is initial triage decisions, where a closed fracture may be managed conservatively with a splint, with the subsequent decision to operate made only at a later follow-up appointment. These "front-end" issues are a key contributor to delays, accounting for 28% (5 of 17) of non-compliant cases, which highlights the critical need for a multidisciplinary team (MDT) approach and targeted educational initiatives [5,12].

The A&E team is vital for correct initial triage and timely referral. By providing structured teaching sessions for A&E and MIU staff, we can aim to improve their ability to identify complex injuries that require immediate specialist referral. This early, expert oversight from a specialist team can prevent incorrect initial management decisions, such as delaying surgery for unstable fractures. This MDT collaboration is essential for a seamless and efficient patient journey, minimising both front-end and back-end delays.

Surgical capacity and staffing

Once a surgical decision is made, the primary bottleneck becomes theatre capacity. This accounted for 52% (9 of 17) of all delays, particularly due to limited access to emergencies or dedicated trauma lists. To address this, the 52 Pre-Op clinic and MIU play a vital role, as their significant utilisation demonstrates the potential for optimisation by expanding their capacity and scope.

Strategic interventions from the QIPS discussion

Based on the audit findings and departmental Quality Improvement and Patient Safety (QIPS) discussions, the following strategic interventions were prioritised:

Establish Regular Rolling Audits

Implement a programme of frequent, smaller-scale audits (e.g., monthly) with robust, standardised documentation to continuously monitor compliance, identify new bottlenecks promptly, and maintain improvement momentum.

Expand Capacity in 52 Pre-Op and MIU

Increase the number of available slots and procedural capabilities within the dedicated Daily Plastics Trauma Clinic (52 Pre-Op) and empower selected Minor Injuries Units (MIUs) within the trust to manage more complex minor hand trauma under protocol, facilitating prompt intervention for appropriate cases closer to presentation.

Implement Educational Outreach to Referring Units

Develop and deliver targeted education sessions (e.g., workshops, guidelines, digital resources) for staff in emergency departments, minor injury units, primary care, and occupational health services, focusing on the critical importance of timely referral for specific hand injuries and the correct referral pathways to plastic surgery.

Introduce Seasonal Theatre Planning

Proactively plan theatre capacity, particularly dedicated trauma lists, to accommodate predictable seasonal increases in hand trauma frequency (e.g., summer months with more outdoor activities, DIY, and gardening) [13]. This aligns with best practices for quality improvement and service redesign [14].

Limitations

This audit acknowledges several limitations. The analysis did not account for seasonal variations in presentations, which typically increase during summer months, potentially skewing the representativeness of the three-month data. The study did not capture patients treated exclusively in emergency departments without specialist follow-up, potentially underestimating the total caseload and the full scope of compliance. Additionally, compliance calculations were based on treatments deemed sufficient by the audit team, potentially introducing subjective bias in certain complex cases.

Future efforts

These combined interventions, expanding dedicated trauma capacity (especially during peaks), optimising use of 52 Pre-Op/MIU, improving referral pathways through education, and implementing continuous monitoring via rolling audits, are crucial for achieving optimal timeliness across all injury types, particularly complex fractures. This multi-faceted approach is essential for enhancing patient care, functional recovery, and effectively minimising the substantial clinical and economic burden (£287 million annually) of hand trauma on individuals and the NHS [1,2,12].

## Conclusions

This second cycle of the hand trauma management audit at UHCW demonstrates commendable progress, achieving an overall 83% (82 of 99 cases) compliance with BSSH surgical timing guidelines, surpassing the 80% target and improving upon the first cycle's 73%. This reflects dedicated efforts in managing a high volume of accidental hand injuries across various settings, aligning with national prevalence data. 

However, significant challenges persist in achieving optimal timeliness for complex injuries. Compliance remains suboptimal for open fractures (64% [9 of 14 cases]) and closed fractures requiring fixation (67% [16 of 24 cases]), and overall (50% [7 of 14 cases]), highlighting systemic bottlenecks within the hand trauma pathway. Continued focus on adhering to national guidelines is paramount to ensure optimal patient outcomes and minimise the substantial functional and economic burden of these injuries.
